# Renal Dysfunction Is an Independent Risk Factor for Mortality after Liver Resection and the Main Determinant of Outcome in Posthepatectomy Liver Failure

**DOI:** 10.1155/2013/875367

**Published:** 2013-11-05

**Authors:** M. G. Wiggans, G. Shahtahmassebi, M. J. Bowles, S. Aroori, D. A. Stell

**Affiliations:** ^1^Hepatobiliary Surgery, Plymouth Hospitals NHS Trust, Derriford Hospital, Derriford Road, Plymouth, Devon PL6 8DH, UK; ^2^Peninsula College of Medicine and Dentistry, University of Exeter and Plymouth University, John Bull Building, Plymouth, Devon PL6 8BU, UK; ^3^School of Science and Technology, Nottingham Trent University, Nottingham NG1 4BU, UK

## Abstract

*Introduction*. The aim of this study was to assess the interaction of liver and renal dysfunction as risk factors for mortality after liver resection. *Materials and Methods*. A retrospective analysis of 501 patients undergoing liver resection in a single unit was undertaken. Posthepatectomy liver failure (PHLF) was defined according to the International Study Group of Liver Surgery (ISGLS) definition (assessed on day 5) and renal dysfunction according to RIFLE criteria. 90-day mortality was recorded. *Results*. Twenty-three patients died within 90 days of surgery (4.6%). The lowest mortality occurred in patients without evidence of PHLF or renal dysfunction (2.7%). The mortality rate in patients with isolated PHLF or renal dysfunction was 20% compared to 45% in patients with both. Diabetes (*P* = 0.028), renal dysfunction (*P* = 0.030), and PHLF on day 5 (*P* = 0.011) were independent predictors of 90-day mortality. *Discussion*. PHLF and postoperative renal dysfunction are independent predictors of 90-day mortality following liver resection but the predictive value for mortality is significantly higher when failure of both organ systems occurs simultaneously.

## 1. Introduction

Despite advances in both operative technique and perioperative care liver resection is associated with mortality rates of 0 to 22% (median 3.7%) and morbidity rates of 12.5% to 66% (median 36%) [1] including liver [2, 3] and renal dysfunction [4]. Liver dysfunction is a major contributor to both morbidity and mortality with an incidence between 1.2% and 32% in published series [5–12]. Renal dysfunction has also been shown to be associated with mortality following liver resection [13], with a reported incidence between 5 and 15% [4, 14]. Posthepatectomy renal failure may occur in conjunction with liver failure when maldistributive circulatory changes occur causing intravascular hypovolaemia [4, 15] but is also related to operative stress and blood loss [16, 17].

Postoperative liver dysfunction has been defined by the “50-50 criteria” as a prothrombin index of less than 50% (mean normal prothrombin time (PT) divided by patient's observed PT) and a serum bilirubin of >50 *μ*mol/L on the fifth postoperative day, which has been shown to predict liver failure and death after hepatectomy [2]. More recently posthepatectomy liver failure (PHLF) has been defined by the International Study Group of Liver Surgery (ISGLS) as a postoperatively acquired deterioration in the ability of the liver to maintain its synthetic, excretory, and detoxifying functions, characterized by an increased INR (or need of clotting factors to maintain normal INR) and hyperbilirubinaemia on or after postoperative day five [18]. The ability of this newer definition of PHLF, using lower measures of dysfunction, to predict mortality has not been thoroughly assessed.

The aim of this study was to assess the utility of the ISGLS definition of PHLF on postoperative day 5 as a predictor of mortality and to determine the interaction of liver and renal dysfunction in predicting 90-day mortality after liver resection. 

## 2. Materials and Methods

A retrospective analysis of a prospectively maintained database of all patients undergoing liver resection in this unit between July 2005 and September 2012 was undertaken. Five hundred and one patients were studied. Patient characteristics, laboratory data, and intraoperative details were retrieved. Liver resections were defined according to the Brisbane classification [19] and undertaken using standard techniques. Prior to resection the operating surgeon makes a visual assessment of the condition of the liver parenchyma and records this as normal or abnormal. Hepatic inflow occlusion was used in a minority of cases where there was excessive blood loss. The POSSUM scoring system was used to calculate the preoperative physiological risk score [20]. 

All patients were followed up for a minimum of 90 days and mortality was recorded along with details of the cause of death. The cause of death was determined from case-sheet review, radiological and laboratory data, and death certificates. Patients who died with jaundice and/or radiological evidence of ascites and/or encephalopathy in the absence of any other clear diagnosis were determined to have died of liver failure. Patients who died within 24 hours of surgery were excluded from further analysis as these deaths were most likely due to perioperative complications. Patients were also excluded if no postoperative blood tests were available.

Serum biochemistry tests and coagulation assays were performed on patients in the first 24 postoperative hours and the tests repeated according to clinical course. The peak measurement of bilirubin, prothrombin time (PT), and creatinine were recorded and used for analysis and patients with PHLF were identified as having an increased PT and serum bilirubin on postoperative day five according to the ISGLS definition [18]. In patients with preoperatively increased PT or serum bilirubin concentration PHLF was defined as an increasing serum bilirubin concentration and increasing PT on postoperative day 5 compared with the values of the previous day. It was not necessary to administer clotting factors to any surviving patients between postoperative days (POD) 1–5. Renal dysfunction was defined as an increase in serum creatinine of ≥1.5-fold from the preoperative baseline within the first five postoperative days, according to RIFLE criteria [21].

To determine potential associations between patient characteristics, operative factors, and organ dysfunction with 90-day mortality univariate logistic regression or chi-square test at the level of *P* < 0.25 [22] was performed, as appropriate. Significant variables in the univariate analysis were included in the multivariate logistic regression model and were considered to be significant if *P* < 0.05. Mortality ratios for organ failure were calculated as the proportion of deaths to proportion of survivors. All analyses were carried out using the statistical package R 2.1.14 [23]. 

## 3. Results

Five hundred one patients were studied. The indications for surgery and preoperative and operative details are shown in Table 1. Two patients who died within 24 hours of surgery were excluded from further analysis. One patient died of heart failure after a partially extended right hepatectomy and one died of biliary sepsis and multiorgan failure following an extended right hepatectomy for hilar cholangiocarcinoma. Details of twenty-one patients (4.6%) who died within 90 days of surgery are shown in Table 2. There was no significant difference in the median age of patients who died (71 years) and those who survived (65 years). The median interval to death after surgery was 31 days (7–89 days). 

Of the 499 patients studied, blood tests were available in 495 patients (99.2%). Four patients did not have postoperative blood tests, all of whom had minor resections (fewer than three segments) and none of whom died within the study period and were excluded from analysis. A summary of liver and renal function tests in the whole cohort is shown in Table 3 along with the associated mortality. 

PHLF occurred in 31 patients of whom two had preexisting liver failure and 12 had extended resections. Seven patients in this group died within 90 days of surgery. Renal dysfunction also occurred in 31 patients, of whom 11 had extended resections. Seven patients in this group died within 90 days of surgery. In 55 patients with diabetes mellitus renal dysfunction occurred in seven patients (12.7%) compared to 24 of 440 patients without diabetes (5.5%) (*P* = 0.067). No patient with diabetes and normal preoperative renal function (*n* = 12) developed postoperative renal dysfunction compared to seven of 43 diabetic patients with impaired preoperative renal function (*P* = 0.326). 

The lowest mortality (2.7%) occurred in the 444 patients without laboratory evidence of PHLF or renal dysfunction at day five, of whom 12 died, compared to 9 of 51 (17.6%) patients with either or both of these diagnoses. In the first group four of the twelve deaths were due to liver failure compared to seven of the nine deaths in the group with evidence of organ dysfunction at POD 5.

The mortality rate in patients who fulfilled the criteria for PHLF on POD 5 but did not have renal dysfunction was identical (2 of 10 patients) to that of patients with renal dysfunction without PHLF (2 of 10 patients). All four of these patients died of liver failure. Mortality was greatest in the group of eleven patients with both PHLF and renal dysfunction of whom five died. Three of these five patients died of liver failure, one from anastomotic leak, and one from a bleeding peptic ulcer.

Multivariate analysis of potential risk factors for mortality including postoperative organ dysfunction (Table 4) revealed that the only preoperative factor independently associated with 90-day mortality was the presence of diabetes (*P* = 0.028), which more than trebled the risk of 90-day mortality. 

Both PHLF on POD 5 and postoperative renal dysfunction were independently associated with 90-day mortality. PHLF at POD 5 increased the risk of 90-day mortality by a factor of 4.5 (*P* = 0.011) and renal dysfunction increased the risk by a factor of 3.6 (*P* = 0.030).

The positive predictive value (PPV) for mortality in patients who fulfilled the criteria for PHLF (including those with and without renal dysfunction) was 22.6%. However within this group the PPV was much lower (10%) if the criteria for PLF were fulfilled with normal renal function (Table 5). The PPV for mortality of fulfilling the criteria for PHLF with concurrent renal dysfunction was 45%. 

**Figure 1:**
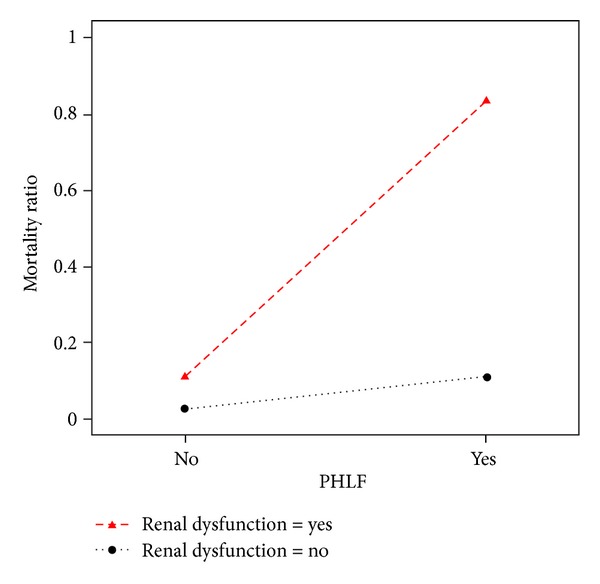
Mortality ratio of combined liver and renal dysfunction in 495 patients undergoing liver resection.

The effect of developing renal dysfunction in the context of PHLF is demonstrated by the greater than fourfold increase in mortality ratio (Figure 1).

## 4. Discussion

The principle findings of this study are that PHLF on POD 5 as defined by the ISGLS and postoperative renal dysfunction are independent predictors of 90-day mortality following liver resection. The predictive value for mortality is significantly higher when failure of both organs occurs, with a PPV of 45% and NPV of 97%. Preoperative diabetes mellitus is also an independent predictor of 90-day mortality.

The 90-day mortality (4.6%) in this series is similar to results of other units [1]. An important observation is that half the postoperative deaths in the series occurred between 31 and 90 days after surgery, stressing the importance of reporting 90-day rather than 30-day mortality. Of the 21 postoperative deaths 11 were found to be due to liver failure. 

The study confirms the ability of PHLF to predict 90-day mortality. Interestingly however the majority of patients who developed PHLF at POD 5 (24 of 31) recovered whilst six of the eleven patients who died of liver failure did not fulfil the ISGLS definition of PHLF at POD 5. Only one patient in this series fulfilled the “50-50 criteria” of postoperative liver dysfunction, who subsequently recovered. Therefore the “50-50” criteria had no value as a predictor of liver failure or mortality in this series with a PPV of zero. In comparison the ISGLS definition of PHLF has lower thresholds for abnormal bilirubin and PT and is a more clinically useful tool for the prediction of 90-day mortality with a PPV of 23% and NPV 97%. This is similar to the findings of the only other study to address this issue, which revealed that the PPV and NPV of PHLF were 32% and 98%, respectively [24]. Simple blood tests therefore have a low positive predictive value for mortality due to liver failure.

Renal dysfunction occurred in 6.3% of patients which is similar to other published series [4, 14]. Renal dysfunction following liver resection may occur as a consequence of liver failure and hepatorenal syndrome but may also result from hypovolaemia or damage from inflammatory mediators during surgery [4]. This occurs more commonly in elderly patients with atherosclerosis or hypertension [15]. These mechanisms of renal dysfunction may occur simultaneously. The use of low central venous pressure (CVP) during resection may also increase the risk of postoperative renal dysfunction [25, 26]. The results of this study demonstrate that isolated renal dysfunction is a significant risk factor for mortality independent of the development of PHLF. Interestingly the two patients with isolated renal dysfunction in the first five postoperative days subsequently died of liver failure. This may be attributed to renal dysfunction delaying the onset of hepatic regeneration [27]. The most marked mortality effect of renal dysfunction was seen in conjunction with PHLF, where the mortality rate increased by a factor of four. Therefore, although the ISGLS definition of PHLF is able to predict mortality due to liver failure the development of renal dysfunction in this context is the single most important predictive factor. 

The finding of the significance of diabetes as a risk factor for postoperative mortality confirms earlier findings [28]. Insulin is important for hepatic function and regeneration [29] and diabetes is also a risk factor for the development of nonalcoholic fatty liver disease and cirrhosis [30] which may lead to higher rates of PHLF [31]. Diabetic nephropathy is also a major cause of renal dysfunction [32]. 

In conclusion we have demonstrated that PHLF as defined by the ISGLS on postoperative day five and postoperative renal dysfunction are able to predict 90-day mortality following liver resection, although most patients fulfilling these criteria of organ dysfunction will recover. In addition many patients will succumb to liver failure without fulfilling the PHLF criteria in the early postoperative period. The combination of these two markers of organ dysfunction is the best early predictor of mortality following liver resection and we suggest that PHLF and postoperative renal dysfunction should be used in conjunction when predicting mortality after liver resection.

## Figures and Tables

**Table 1:** Preoperative and intraoperative characteristics of 501 patients undergoing hepatic resection.

n = 501	Median (range)	Count (%)
Age	65 (21–90)	
Gender		
Female		223 (45)
Male		278 (55)
Indication for surgery		
Benign		46 (9)
Primary		
Hepatocellular carcinoma		39 (8)
Cholangiocarcinoma		31 (6)
Others		28 (6)
Secondary		
Colorectal metastases		308 (61)
Other metastases		49 (10)
Liver directed chemotherapy		
Yes		176 (35)
No		325 (65)
Diabetes		
Yes		55 (11)
No		446 (89)
BMI	26 (16–54)	
ASA Grade		
1		51 (10)
2		323 (64)
3		124 (25)
4		2 (0.4)
Not recorded		1 (0.2)
Physiologic risk score	16 (12–32)	
Operative risk score	24 (14–35)	
Estimated P-POSSUM mortality (%)	7.7 (0.9–69.3)	
Confirmed fibrosis/cirrhosis		
Yes		22 (4)
No		479 (96)
Preoperative bilirubin (*µ*mol/L)	9 (2–162)	
Preoperative haemoglobin (g/dL)	13.2 (8.6–17.0)	
Preoperative white cell count (/L)	6.9 (2.7–25.0)	
Preoperative albumin (g/L)	44 (24–53)	
Preoperative alkaline phosphatase (U/L)	95 (34–1190)	
Preoperative creatinine (*µ*mol/L)	78 (40–430)	
Preoperative glomerular filtration rate (GFR)		
>90 mL/min		163 (33)
<90 mL/min		326 (65)
Not measured		12 (2)
Preoperative neutrophil lymphocyte ratio (NLR)	2.5 (0.3–17.3)	
NLR > 5		
Yes		59 (12)
No		442 (88)
Open or laparoscopic approach		
Open		453 (90)
Laparoscopic		48 (10)
Radio frequency ablation (RFA) included		
Yes		23 (5)
No		478 (95)
Wedge resection included		
Yes		189 (38)
No		312 (62)
Operation		
Right hemihepatectomy		173 (35)
Extended right hemihepatectomy		34 (7)
Left hemihepatectomy		64 (13)
Extended left hemihepatectomy		17 (3)
Left lateral sectorectomy		48 (10)
Wedge resection only		133 (27)
Other		32 (6)
Bile duct reconstruction included		
Yes		46 (9)
No		455 (91)
Synchronous bowel procedure		
Yes		23 (5)
No		478 (95)
Operation number		
1st resection		465 (93)
2nd resection		31 (6)
3rd resection		5 (1)
Number of segments resected	4 (1–6)	
Number of procedures	1 (1–10)	
Surgeon's assessment of liver parenchyma		
Normal		323 (64)
Abnormal		171 (34)
Not recorded		7 (1)
Blood loss		
<500 mL		246 (49)
500–999 mL		175 (35)
≥1000 mL		76 (15)
Not recorded		4 (0.8)
Units transfused	0 (0–26)	

**Table 2:** Details of 21 patients who died within 90 days of surgery. (Two patients who died within 24 hours of surgery were excluded.)

Cause of death	Count	Gender		Age	Right hepatectomy	Extended right	Extended left	Minor resection	Interval to death (days)
		Male	Female						
Liver failure	11	9	2	67 (58–76)	3	7	1	0	31 (11–83)
Malignancy	4	2	2	58 (43–76)	2	1	0	1	68.5 (14–86)
Sepsis	1	1	0	71	0	1	0	0	15
PE	1	1	0	71	1	0	0	0	7
Anastomotic leak	1	1	0	80	0	0	0	1	8
Peptic ulcer	1	0	1	81	1	0	0	0	22
Strangulated hernia	1	1	0	76	0	0	0	1	89
Peritonitis	1	1	0	76	0	0	0	1	70

**Table 3:** Postoperative liver and renal dysfunction in 495 patients undergoing hepatic resection (blood tests not performed in four patients).

Laboratory parameters at day 5 (*n* = 495)	Count (%)	90-day mortality (%)	Death due to liver failure
No PHLF or renal dysfunction	444 (89.7)	12 (2.7)	4
PHLF alone	20 (4.0)	2 (10)	2
Renal dysfunction alone	20 (4.0)	2 (10)	2
Renal dysfunction plus PHLF	11 (2.2)	5 (45.5)	3

**Table 4:** Univariate and multivariate analysis of preoperative and operative factors as well as postoperative blood tests associated with 90-day mortality following liver resection in 495 patients.

*n* = 495	Univariate		Multivariate	
Factor (preoperative and operative factors and postoperative blood tests)	Coef (95% CI)	*P* value	Coef (95% CI)	*P* value
Age	1.05 (1.01–1.10)	0.029*		0.194
Gender	2.36 (0.91–6.08)	0.077*		0.196
Pathology		0.274		
Liver directed chemotherapy		0.356		
Diabetic	3.09 (1.16–8.20)	0.024*	3.41 (1.14–10.23)	0.028**
BMI		0.444		
ASA grade				
1 versus 2	3.02 (0.70–13.11)	0.139*		0.678
2 versus 3		0.724		
Physiologic score	1.12 (1.03–1.22)	0.010*		0.544
Operative score		0.303		
P-POSSUM mortality	1.04 (1.01–1.07)	0.010*		0.479
Fibrosis/cirrhosis		0.986		
Preoperative bilirubin	1.01 (1.00–1.03)	0.081*		0.652
Preoperative haemoglobin	0.71 (0.55–0.93)	0.012*		0.195
Preoperative white cell count		0.388		
Preoperative albumin	0.90 (0.84–0.96)	0.002*		0.168
Preoperative alkaline phosphatase		0.884		
Preoperative creatinine	1.01 (1.00–1.02)	0.098*		0.764
Preoperative neutrophil lymphocyte ratio	1.13 (0.98–1.31)	0.086*		0.366
Preoperative neutrophil lymphocyte ratio >5	2.18 (0.78–6.11)	0.138*		0.345
Open or laparoscopic resection		0.987		
Radiofrequency ablation (RFA) included		0.991		
Wedge resection included		0.588		
Bile duct reconstruction included	2.96 (1.05–8.39)	0.041*		0.383
Synchronous bowel procedure		0.346		
Operation number		0.549		
Number of segments resected	1.59 (1.18–2.14)	0.003*		0.075
Number of procedures		0.786		
Surgeons assessment of liver parenchyma	2.14 (0.92–4.96)	0.076*		0.494
Blood loss (mL)				
<500 versus >500	2.67 (1.27–5.61)	0.009*		0.716
>500 versus >1000		0.652		
Units of red cells transfused	1.13 (1.02–1.26)	0.023*		0.224
PHLF at POD 5	1.02 (1.01–1.03)	<0.001*	4.51 (1.42–14.40)	0.011**
Renal dysfunction (creatinine rise >1.5x)	1.02 (1.01–1.03)	<0.001*	3.63 (1.13–11.66)	0.030**

*Significant at the level of 0.25 for univariate analysis and included in multivariate analysis.

**Significant at the level of 0.05 for multivariate analysis.

**Table 5:** Predictive values of PHLF and renal dysfunction within the first five postoperative days in 495 patients undergoing liver resection.

	Positive predictive value (PPV)	Negative predictive value (NPV)
No PHLF or renal dysfunction	0.027	0.824
PHLF alone	0.1	0.970
Renal dysfunction alone	0.1	0.970
PHLF and renal dysfunction	0.455	0.967
